# A Biophysical Systems Approach to Identifying the Pathways of Acute and Chronic Doxorubicin Mitochondrial Cardiotoxicity

**DOI:** 10.1371/journal.pcbi.1005214

**Published:** 2016-11-21

**Authors:** Bernardo L. de Oliveira, Steven Niederer

**Affiliations:** Department of Biomedical Engineering, Division of Imaging Sciences and Biomedical Engineering, King’s College London, London, United Kingdom; University of Michigan, UNITED STATES

## Abstract

The clinical use of the anthracycline doxorubicin is limited by its cardiotoxicity which is associated with mitochondrial dysfunction. Redox cycling, mitochondrial DNA damage and electron transport chain inhibition have been identified as potential mechanisms of toxicity. However, the relative roles of each of these proposed mechanisms are still not fully understood. The purpose of this study is to identify which of these pathways independently or in combination are responsible for doxorubicin toxicity. A state of the art mathematical model of the mitochondria including the citric acid cycle, electron transport chain and ROS production and scavenging systems was extended by incorporating a novel representation for mitochondrial DNA damage and repair. In silico experiments were performed to quantify the contributions of each of the toxicity mechanisms to mitochondrial dysfunction during the acute and chronic stages of toxicity. Simulations predict that redox cycling has a minor role in doxorubicin cardiotoxicity. Electron transport chain inhibition is the main pathway for acute toxicity for supratherapeutic doses, being lethal at mitochondrial concentrations higher than 200*μM*. Direct mitochondrial DNA damage is the principal pathway of chronic cardiotoxicity for therapeutic doses, leading to a progressive and irreversible long term mitochondrial dysfunction.

## Author Summary

Doxorubicin is a potent anticancer drug, but its efficacy is limited by a cumulative dose-dependent cardiotoxicity. Multiple pathways are involved in the drug cardiotoxicity, however, the underlying mechanisms are still not fully elucidated. Here we developed a computational model to study doxorubicin mitochondrial cardiotoxicity, which allowed for the first time, a systematic test of different hypothesis in a unified framework. By quantitatively comparing the effect of multiple toxicity mechanisms, we could identify that electron transport chain is the main cause of acute toxicity, while direct damage to the mitochondrial DNA is the principal pathway of chronic cardiotoxicity. This is a crucial step in developing new antitumor therapies, toxicity screens and developing treatments to mitigate doxorubicin cardiotoxicity.

## Introduction

Doxorubicin (DOX) is an anthracycline antibiotic with potent antineoplastic properties [1]. It has a broad-spectrum and is widely prescribed in the treatment of many types of cancers, including solid tumors and leukemias [2]. Yet, the clinical use of this drug is restricted by its severe side effects. DOX presents dose dependent, cumulative and irreversible cardiotoxicity that can lead to cardiomyopathy and ultimately congestive heart failure [3]. However, the underlying biochemical mechanisms of its toxicity are still not fully elucidated.

Different processes are involved in DOX cardiotoxicity, including apoptosis, intracellular calcium dysregulation and myofibrillar detereoration, among others [4]. DOX cardiotoxicity is also strongly associated to mitochondrial dysfunction that leads to increased reactive oxygen species (ROS) production and cardiac oxidative stress [5]. DOX can inhibit the electron transport chain (ETC) by binding to cardiolipin which is present in the inner mitochondrial membrane [6]. As cardiolipin is required for normal ETC activity, this interaction leads to ETC inhibition [7]. During acute exposure, DOX also increases ROS production by undergoing redox cycling. The drug is capable of oxidising Complex I of the ETC, stealing electrons and transfering them directly to oxygen, producing ROS [8]. Furthermore, DOX acts as a topoisomerase II poison [9] and can form DNA adducts [10], which can damage the DNA and inhibit gene transcription and DNA replication [11–14].

**Fig 1:**
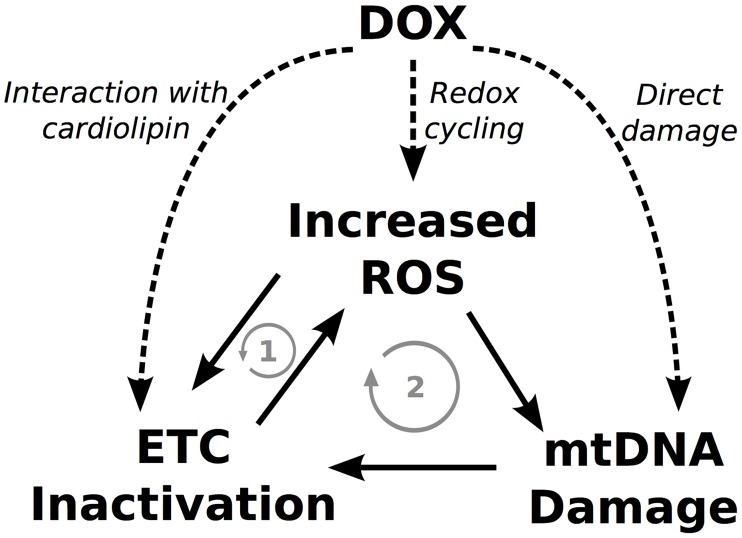
DOX could trigger vicious cycles that lead to progressive mitochondrial dysfunction. The dashed arrows represent the acute effects of DOX. The solid arrows represent the interactions between elements while the gray arrows represent the potential vicious cycles that could be formed.

It has been suggested that these interactions can form vicious cycles that could continue operating even after the termination of the treatment, accumulating over time, and ultimately leading to bioenergetic failure [15]. DOX can potentially influence all elements of these cycles as depicted in Fig 1 where two possible vicious cycles can be observed. Vicious cycle one involves increased ROS levels which can inactivate the ETC and cause a further increase in ROS production. In vicious cycle two, increased ROS levels cause mtDNA damage which can lead to a downregulation of the ETC proteins that are mtDNA encoded, exacerbating mitochondrial dysfunction and ROS production.

The objective of this study is to test the hypothesis that such vicious cycles could be formed by developing a computational model that quantitatively links alterations in ETC activity, ROS production and mtDNA damage with mitochondrial dysfunction. The goal is to quantify the contributions of each of these different toxicity pathways and possible vicious cycles to mitochondrial dysfunction associated with acute and chronic DOX cardiotoxicity.

## Results

### Acute Effect

To quantify the relevance of vicious cycle one depicted in Fig 1, the acute effects of DOX were initially studied taking only ETC inhibition and redox cycling into account. This allowed us to investigate these two mechanisms in isolation and combined, prior to accounting for mtDNA damage.

**Fig 2:**
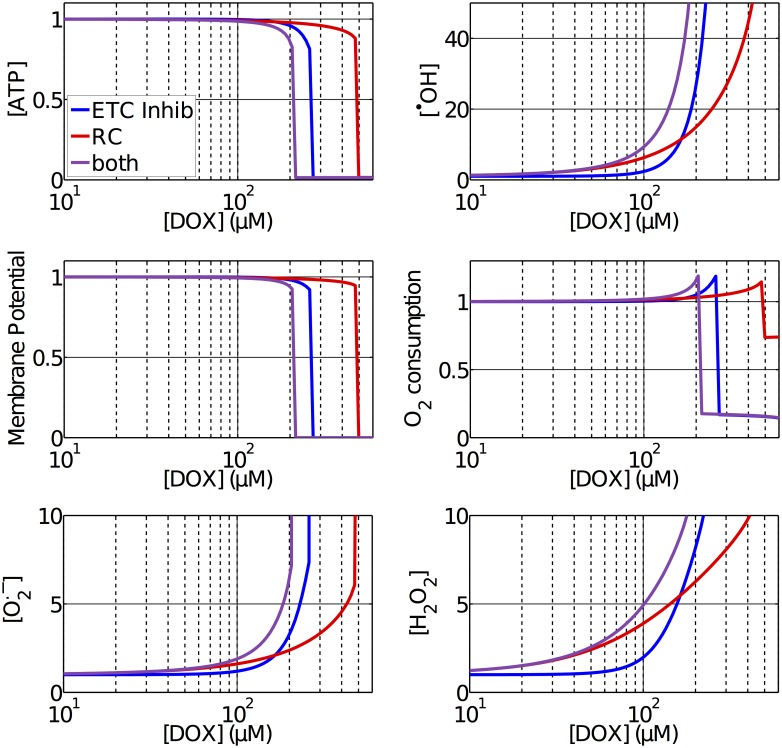
Normalized variation of different indices of mitochondrial function with respect to acute concentrations of DOX in the mitochondria. A constant concentration of DOX was used as an input and simulations were performed until steady state. It is possible to compare the effects of ETC inhibition, redox cycling (RC) and both combined.

The predicted acute effects of redox cycling and ETC inhibiton at different concentrations of DOX can be observed in Fig 2. This experiment consisted of introducing a constant concentration of the drug and performing a simulation until the model reached a steady state. This allowed us to quantify how mitochondrial function varies in the presence of different concentrations of the drug.

Across all simulations we can observe similar features. For low drug concentrations, the ATP concentration and the membrane potential were only barely reduced, with an associated increase in the *O*_2_ consumption and concentrations of [^•^*OH*], $\left[O_{2}^{.-}\right]$ and [*H*_2_*O*_2_]. We can also observe that, for concentrations of up to 160*μM*, redox cycling is the main contributor for the increase in ROS, while for concentrations higher than that, the effect of ETC inhibition becomes dominant. For high drug concentrations, mitochondrial function gradually deteriorate until a threshold is reached and the mitochondria completely collapse. This causes a complete loss of membrane potential and ATP concentration, a sharp increase in the ROS concentrations and a reduction in the *O*_2_ consumption to residual levels, indicating that the dose may be lethal. This threshold happened at 480*μM* in the simulations including redox cycling only, 270 *μM* taking only ETC inhibition into account and 210 *μM* taking both into account.

**Fig 3:**
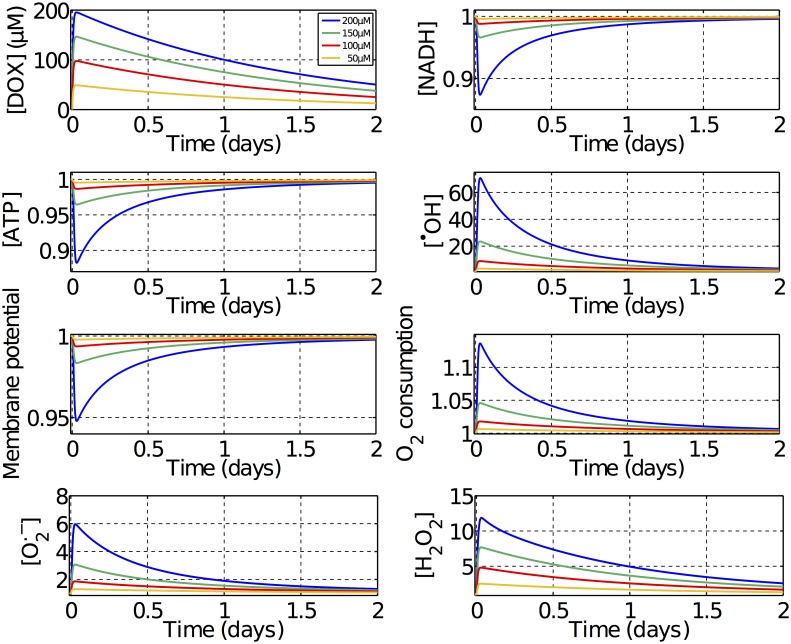
Normalized dynamic variation of mitochondrial function to different doses of DOX. Time varying concentrations of DOX following the drug’s pharmacokinetics were used as an input. The resulting variation in different measurements of mitochondrial function can be observed.

A series of dynamic simulations were performed to investigate if ETC inhibition and redox cycling could lead to any permanent alteration in mitochondrial function by forcing the mitochondria into a new steady state. In these simulations, time varying concentrations of DOX were used as an input to the model, respecting the drug pharmacokinectics. A fast absorption of the drug was assumed with half-time of 5 minutes while the elimination of the drug was considered to be slower with a half-time of 24 hours [16] as depicted in Fig 3. Four supratherapeuthic doses were tested spanning a range of concentrations lower than the lethal dose of 210 *μM*, that was predicted from the simulations presented in Fig 2. For low doses, mitochondrial function is only marginally affected, but at high doses, some significant variations can be observed. Mitochondrial function deteriorates as the dose increases, with a decrease in ATP concentration, membrane potential and NADH levels and an increase in ROS concentrations and *O*_2_ consumption. Additional simulations revealed that decreases in both the membrane potential and matrix pH equally contribute to an alteration in Complex IV activity which results in the increased *O*_2_ consumption observed. In all the dynamic simualtions, these effects are always temporary and all quantities return to their baseline values after the drug is fully eliminated from the system. This indicates that ETC inhibition and redox cycling, and thus the possible vicious cycle one in Fig 1, are not sufficient to explain long term mitochondrial dysfunction associated with DOX.

### Chronic Effect

As our model including redox cycling and ETC inhibition was not capable of reproducing any permanent and long term alteration in mitochondrial function, the mtDNA model represented in Eq 1 was introduced to investigate if mtDNA damage, and vicious cycle two in Fig 1, could explain the chronic toxicity of DOX.

**Fig 4:**
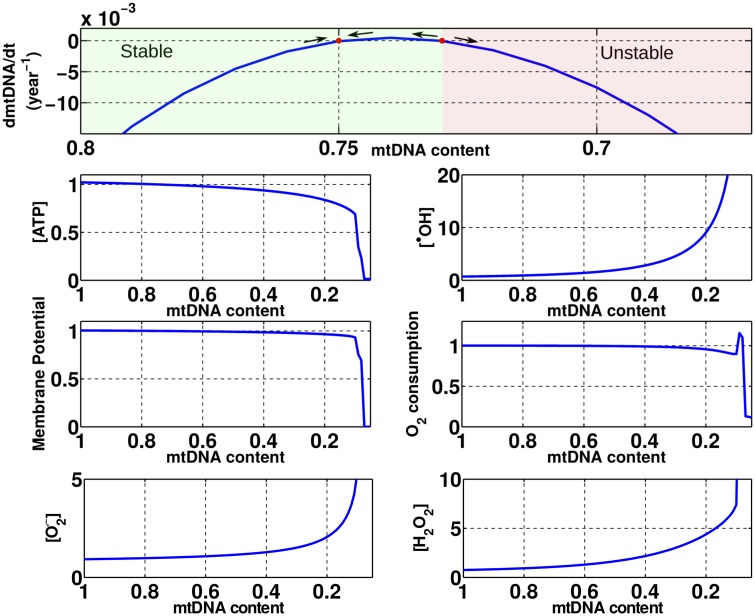
Effects of the variation of the mtDNA content in mitochondrial function. It is possible to observe a bifurcation point when the mtDNA content is equal to 0.73. For mtDNA contents higher than 0.73 the mitochondria are in a stable condition and recovers to baseline over time. For a mtDNA content lower than 0.73, the mitochondria are unstable and its function will perpetually deteriorate until collapse.

Prior to investigating the damaging effects of DOX to the mtDNA, we performed simulations to evaluate how mitochondrial function is affected by variations in the mtDNA content without including any DOX effect. In our model, as the mtDNA content is altered, the expression of all proteins and enzymes encoded by it are scaled, namely, Complexes I, III, IV and ATP synthase. In order to generate a phase plot and to predict the dependency of mitochondrial function on the mtDNA content, multiple experiments were performed by holding the mtDNA content constant at different values and simulating until a new steady state was achieved. The results of these simualtions can be seen in Fig 4. We can observe that at baseline conditions with a mtDNA content of 0.75, its time derivative, *dmtDNA*/*dt*, is equal to zero, in a stable condition with all the indices of mitochondrial function also at baseline.

For mtDNA contents higher than baseline, no significant improvement in mitochondrial function is observed and *dmtDNA*/*dt* is negative. This is caused by an attenuation of the mtDNA repair activity such that intrinsic ROS production causes enough damage to mtDNA to reduce its content to baseline conditions over time.

For mtDNA contents lower than baseline, *dmtDNA*/*dt* has a biphasic behaviour with a positive and a negative region. A stable region is observed for mtDNA contents between 0.75 and 0.73. In this region, *dmtDNA*/*dt* is positive, as the mtDNA repair activity is greater than the mtDNA damage caused by ROS, causing the mtDNA content to recover back to baseline. An unstable region is observed for mtDNA contents lower that 0.73. In this region, *dmtDNA*/*dt* is negative, as the increased ROS concentrations generate more mtDNA damage than the mtDNA repair system can handle. This indicates that 0.73 is a bifurcation point that is a threshold of how much mtDNA damage a mitochondrion can recover from. Any reduction in mtDNA content below 0.73 leads to a perpetuating and progressive decrease in mitochondrial function.

It is also possible to observe that reductions in the mtDNA content also lead to an increase in the $O_{2}^{.-}$ concentration. As the mtDNA content is reduced, $O_{2}^{.-}$ production by Complex I is reduced, but a concomitant increase in $O_{2}^{.-}$ production by Complex III is observed and the combined $O_{2}^{.-}$ production monotonically increases. The superoxide production by Complex III is increased as a reduction in the density of this complex leads to a reduction in the rate of the reactions involved in the Q-cycle. This causes changes in the concentrations of the substrates involved in these reactions, including an increase in the semiquinone radical ion concentration. This increase in the semiquinone radical ion concentration consequently leads to an increase in the rate that this radical is oxidized by *O*_2_, which is the source of $O_{2}^{.-}$ production by Complex III.

**Fig 5:**
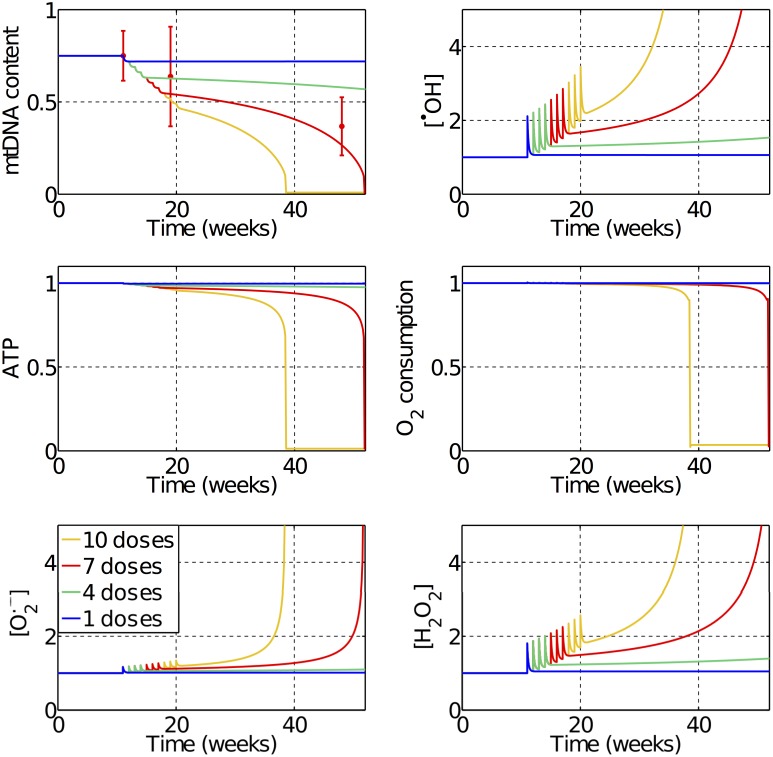
Predicted effects of the treatment with weekly doses of 30*μM* of DOX. One dose is not sufficient to trigger the vicious cycle responsible for chronic cardiotoxicity. With four doses, the vicious cycle is triggered but the progression is slow. With seven doses, a faster progression is observed and the predicted reduction in mtDNA content is within the error of the experimental data used to fit the model’s parameters [15]. With ten doses a fast deterioration and collapse in mitochondrial function is observed.

Fig 5 shows the model predictions for how mitochondria function is affected over time by different numbers of weekly doses of 1mg/kg of DOX, which are equivalent to doses of 30*μM* in our model. The red errorbars in the first panel are the experimental data points used to fit the model parameters [15], and are related to in vivo measurements of mtDNA content after seven weekly doses of DOX in rats. With only one dose, we can already observe long term alterations in mitochondrial function, however, the mtDNA content is only slightly reduced and the mitochondria manage to recover through mtDNA repair. With four doses the damage is already large enough to trigger a vicious cycle, but the progression of the mitochondrial dysfunction is slow as the damage is relatively small and may not lead to observable symptoms. With seven doses and more, the damage is significant, triggering a fast degradation of mitochondrial function. We can observe a progressive reduction in the mtDNA content and ATP concentrations and an increase in ROS levels. The spikes observed in the curves are related to the DOX doses that have a peak while the drug is still in the system, but keeps a cumulative dysfunction even after the drug is eliminated due to the mtDNA damage. Our model also predicts that direct mtDNA damage by DOX is the main pathway that triggers this vicious cycle, being responsible for over 75% of the mtDNA content reduction during the acute stages of DOX intoxication.

### Cardioprotection Simulation

Free iron plays an important role in modulating DOX cardiotoxicity by serving as a catalyst to the formation of hydroxyl radicals through the Haber-Weiss reaction [17]. Iron chelators have demonstrated cardioprotection properties when co-administred with DOX as they bind to iron and eliminate this heavy metal from the body [18, 19]. More specifically, co-administration of Dexrazoxane with DOX has been shown to prevent a rise in free iron levels observed when administring DOX in isolation, reverting this cardiotoxic effect and keeping the the free iron levels at baseline [20].

**Fig 6:**
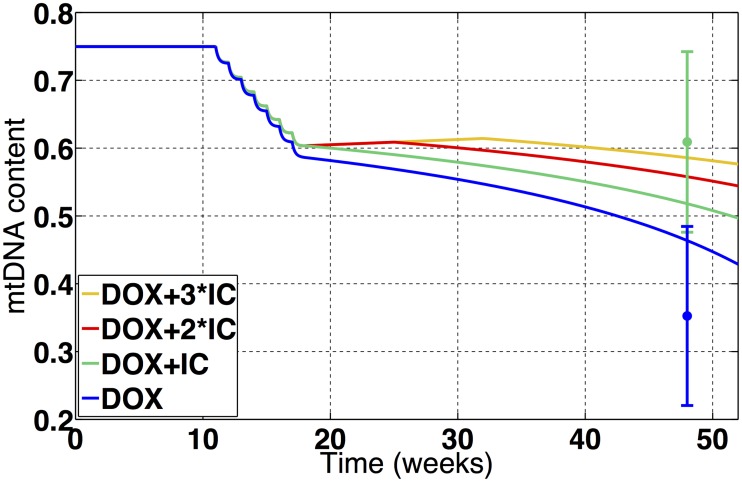
Predicted effects of the treatment with seven weekly doses of 24*μM* of DOX with and without co-treatment with iron chelators. In the simulation without iron chelator co-treatment (blue), the predicted reduction in the mtDNA content agrees with the experimental data, which was used in the fitting of the model’s parameters [21]. The model was capable of capturing the cardioprotective feature of iron chelator co-treatment (green), and the predicted reduction in the mtDNA content also agrees with experimental data [21]. The model predicts that extending the iron chelation therapy by double (red) or triple (yellow) the duration of the DOX treatment can enhance cardioprotection.

Co-administration of Dexrazoxane has also been shown to mitigate mtDNA damage and the loss of mtDNA content associated with DOX [21]. To test if our model is capable of capturing this protective property, we used a simplified model of chelating therapy by assuming that the free iron levels are kept constant at baseline during chelating treatment [20]. A detailed description can be found in the supplemental material. Our simulations reproduced the setup of this in vivo experiment where seven weekly doses of 0.8 mg/kg of DOX, which are equivalent to doses of 24*μM* in our model, were administred in rats, with and without the co-administration of iron chelators, and the mtDNA content was measured 37 weeks after the termination of the treatment [21]. Fig 6 shows the variation in the mtDNA content observed in these simulations, while the errorbars are the mtDNA content measured in the in vivo experiment. We can see that our model was able to capture the protective properties of the chelating therapy, although to a lesser extent than the ones observed in vivo. Our model also predicts that extending the chelating treatment to two of three times the duration of the DOX treatment might considerably increase the cardioprotection offered by further decreasing the loss of mtDNA content.

## Discussion

In this work, a biophysical model of the mitochondria was adapted to represent the cardiotoxic effects of DOX. Computational models have already been used to study mitochondrial dysfunction [22], ROS generation [23, 24] and ROS scavenging [25, 26]. However, this is the first time, that this kind of models has been used to study drug cardiotoxicity.

Three pathways of DOX cardiotoxicity were modeled, with all parameters constrained using experimental data, and their contributions to mitochondrial dysfunction were quantified. Our model predicts that although redox cycling is the main contributor to acute increases in ROS concentrations at clinically relevant concentrations, of approximatelly 30*μM* [16], it has a minor role in DOX cardiotoxicity as any considerable loss of mitochondrial function can only be obeserved at much higher concentrations, as observed in Fig 2. ETC inhibition also showed negligible effects at clinically relevant concentrations, however, when a critical mitochondrial concentration of 210 *μM* is reached, it is the principal mechanism for a sharp and rapid collapse in mitochondrial function. For doses higher than this critial concentration, the mitochondria are not able to sustain the membrane potential, which causes a collapse in mitochonrial function and depletion of ATP. These results are in agreement with experiments where mice treated with a single 15mg/kg dose of DOX, which correspond to a dose of 450*μM* in our model, were used to test the hypothesis of redox cycling mediated cardiotoxicity [14]. This experiment showed a reduction in ETC activity and a rapid depletion of ATP, followed by a decrease in the expression of myocardial ETC genes.

Our results also showed that redox cycling and ETC inhibition alone are not capable of generating any long term alterations in mitochondria function, as depicted in Fig 3. The chronic cardiotoxicity of DOX was only reproducible when taking mtDNA damage into account, which was necessary and sufficient to trigger a vicious cycle that leads to a progressive loss of mitochondrial function. These findings highlight the importance of dosing for in vivo and in vitro experiments when investigating DOX cardiotoxicity as the dominant toxicity pathways of acute therapeuthic dosing, acute supratherapeuthic dosing and chronic therapeuthic dosing could be different.

To study chronic DOX toxicity, a novel mtDNA damage and repair model was proposed, including the subsequent alterations in the expression of mtDNA encoded proteins that was fit to experimental data [15]. This model was capable of reproducing the cumulative and progressive long-term effects of DOX toxicity in the time course of weeks and even years. We observed that the effect of a single clinical dose is not sufficient to lead to progressive mitochondrial dysfunction as the mitochondria manage to recover. However, mtDNA damage accumulates after sucessive doses and vicious cycle 2 depicted in Fig 1 is triggered. With mtDNA damage, the expression of mtDNA encoded proteins is reduced, leading to progressive mitochondria dysfunction until bioenergetic failure. As observed in Fig 4, a mtDNA content reduction of approximately 5% with respect to baseline is enough to trigger a vicious cycle by moving mitochondria function from a stable to an unstable state.

The assumptions made in the model, related to ^•^*OH* production, potentially overestimate oxidative mtDNA damage (see section S3 of the supplemental material for details), however, this is unlikely to alter the study conclusions as we identified direct damage to mtDNA by DOX as the main pathway to trigger the vicious cycle responsible for DOX chronic cardiotoxicity. It was quantified that direct mtDNA damage by DOX is responsible for over 75% of the mtDNA content reduction during the acute stages of intoxication. Although oxidative mtDNA damage by ROS has a secondary role during the acute stages, it allows this vicious cycle to be sustained after the chemotherapy treatment is completed and the drug has been eliminated. These results are in agreement with experiments that showed that cardiomyocite specific deletion of the gene encoding topoisomerase-II*β*, involved in mtDNA damage by DOX, protects cardiomyocytes from doxorubicin induced defective mitochondria and ROS formation [9], while co-administration of ROS scavengers and antioxidants failed to prevent cardiac toxicity both experimentally [27] and clinically [28].

The only approved cardioprotective agent that has shown efficacy when co-administred with DOX in clinical settings is the iron chelator Dexrazoxane [28, 29]. Our model was capable of capturing this protective property of iron chelator co-administration, which reduces the initial insult to mtDNA, as shown in Fig 6. However, this protection is partial, not only because mtDNA damage by ROS has a secondary role during the acute stages, but also because, even when the iron levels are kept at baseline, an increase in the hydroxyl concentration and mtDNA damage by ROS is still observed as a consequence of increased peroxide concentrations. The model predicts that extending the iron chelating therapy to time periods longer than the DOX treatment can enhance this protective property. This generates a longer time period with reduced oxidative damage to mtDNA by ROS, allowing the mitochondria to repair more of the initial damage, potentially reverting the vicious cycle or at least slowing down the progression of dysfunction.

All models are inherently simplifications and aim to represent the salient features of the underlying system. Here we discuss the limiations and assumptions of the models and the potential impact on the study conclusions. The repair systems of mtDNA are complex and still poorly understood, with mulitple mechanisms reported in the literature [30]. Due to the sparsity of experimental data available, a simplified model was adopted, with all mtDNA repair activity lumped into a single enzymatic term. Also, due to the limited data to constrain the model’s parameters, an additional 15,000 simulations were performed, exhaustively exploring the space of potential parameter combinations, to test if the study results were dependent on the specific parameter set evaluated. All of the evaluated parameter combinations, that generated results within the errorbars of the experimental data, support the conclusion that direct damage to mtDNA by DOX is the main toxicity pathway responsible for triggering the vicious cycle that leads to mitochondrial dysfunction. More details can be found in section S3 of the supplemental material.

In the model, the expression of mtDNA encoded proteins was considered to be proportional to the mtDNA content. Although these quantities are correlated, there could be delays between the mtDNA damage and the reduction in the density of mtDNA encoded proteins, and this could play a role especially during the initial stages of the cardiotoxicity. Also, although redox cycling and oxidative damage to mtDNA are represented in our model, we do not take into account oxidative damage to any other structures or proteins. It is possible that the damage caused by the elevated ROS levels to other structures contributes to DOX cardiotoxicity. This may effect proteins, lipids and other pathways not represented in this model, including calcium dysregulation [31] and mitochondrial permeability transition [32]. It has also been proposed that DOX removes proginator cells that may contribute to a heart failure phenotype [33]. These may be contributing factors, however, the observed increase in ROS production and decrease in mtDNA content are consistent with the mitochondria playing a prominent role in DOX cardiotoxicity.

Despite these limitations, this work presents a computational model for DOX mitochondrial cardiotoxicity that gives new insights into the drug’s toxicity mechanisms and cardioprotection alternatives and allows us to combine and evaluate multiple hypothesis concurrently within a common framework. The models developed here can be further used to test different DOX treatment protocols, cardioprotection strategies or to study the cardiotoxicity of other drugs. The framework of this study and the novel mtDNA damage and repair model developed here have applications even beyond drug cardiotoxicity, as mitochondrial dysfunction and mtDNA damage are associated with multiple other pathologies and applications such as heart failure [34], cardiac and cerebral ischemia reperfusion injury [35, 36] and aging [37].

## Models

**Fig 7:**
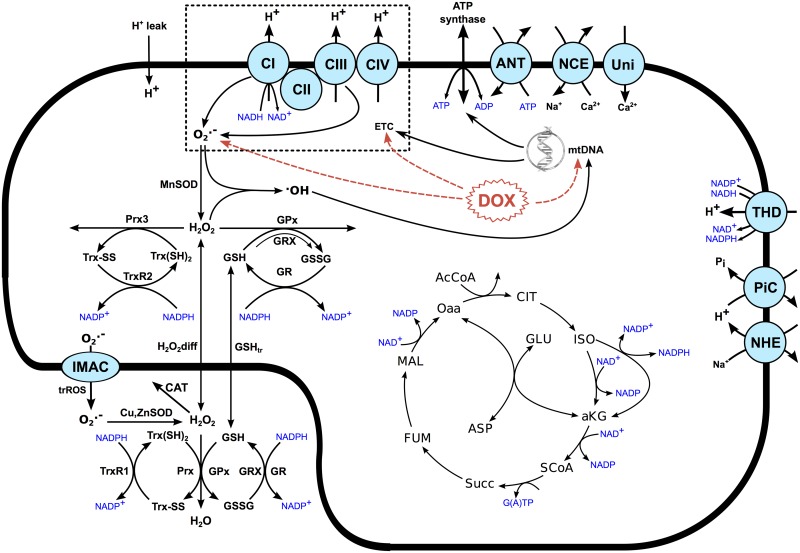
A schematic of the mitochondrial model used. The expressions of ATP synthase and Complexes I, III and IV of the ETC are scaled by the mtDNA content as these structures are encoded at mtDNA. The acute effects of DOX are highlighted in red.

A detailed biophysical computational model of the mitochondria was adapted to simulate the effects of DOX. The original model [38] incorporates, in a unified framework, all the major components for the study of DOX mitochondrial cardiotoxicity: the TCA cycle, transporters, ROS production and scavenging systems and a detailed ETC representation [39]. All simulations in this work consider that the mitochondria are in the presence of substrate and ADP (state 3 respiration), and results are normalized with respect to baseline conditions, which were calculated by simulating the mitochondria in the absence of DOX until steady state. The acute effects of DOX depicted in Fig 1 were modeled and incorporated into the biophysical mitochondria model, shown in Fig 7, where the acute DOX effects are highlighted in red. This section will briefly descibe how each these toxic pathways were modeled. A full description of all the model’s equations, parameters and constants adopted can be found in the supplemental material.

### ETC Inhibition

When present in the mitochondria, DOX binds onto cardiolipin in the mitochondrial membrane which in turn inhibits the complexes of the ETC. The activity of each of the four ETC complexes has been recorded in isolation at multiple concentrations of DOX, and the *IC*_50_ values have been reported in the literature [7]. In our model, this data along with corresponding fitted Hill coefficients, were used to construct dose dependent functions to scale the activity of each of the ETC complexes. More details can be found in section S1 of the supplemental material.

### Redox Cycling

Increased ROS production by redox cycling was represented by augmenting superoxide production by Complex I, which has been identified as the redox cycling site for DOX [8]. This increase in superoxide production was considered to be proportional to the concentration of the drug and fitted to experimental data [15]. In this experiment, a 7% increase in the superoxide concentration was measured two hours after the administration of 1*mg*/*kg* of DOX in rats. In humans, this dose is equivalent to a clinically relevant concentration of 37*mg*/*m*^2^ [40] which generate mitochondrial concentrations in the range of 5 to 30*μM* [16]. In this study, the redox cycling parameters were manually adjusted to generate a similar 7% increase in the superoxide concentration for a DOX dose of 10*μM*. More details can be found in section S2 of the supplemental material.

### Damage to mtDNA

To take into account the damaging effects of DOX in the mtDNA, we propose a new mass action model for mtDNA damage and repair. This model includes a variable for the mtDNA content, which was considered unitless and normalized. At baseline conditions the damaging term is equal to the repair term, keeping the mtDNA content constant at 0.75 [41, 42]. The expression of all proteins and enzymes encoded in mtDNA was considered to be scaled by the mtDNA concentration. More specifically, Complexes I, III, IV and ATP synthase have their expression and protein densities scaled by the mtDNA content. We consider that when mtDNA is damaged, its content is reduced and if mtDNA is repaired, its content is increased as represented in Eq 1:

$$\frac{d(mtDNA)}{dt}=\alpha \cdot \left(\frac{\left(1-mtDNA\right)}{(1-mtDNA)+\kappa }\right)-\beta \cdot {\left[{}^{•}OH\right]}_{n}\cdot mtDNA-\gamma \left[DOX\right]\cdot mtDNA.$$ (1)

Where *α* is the mtDNA repair maximum rate, *κ* is the mtDNA repair half-saturation coefficient, *β* is the coefficient for mtDNA damage by ROS, [^•^*OH*]_*n*_ is the normalized hydroxil radical concentration and *γ* is the coefficient for mtDNA damage by DOX. The first term of the equation represents the mtDNA repair system. As the mtDNA repair activity is conducted by enzymes, this term was considered to have an assymptotic behaviour [30, 43]. If the mtDNA is damaged and its content is decreased, the repair activity increases until a saturation is achieved where the system is working at full power. The second term represents mtDNA damage by [^•^*OH*] which is a ROS capable of damaging DNA [44]. This highly reactive oxidant has been shown to be produced in biological systems through iron-catalyzed Haber-Weiss reaction, which make use of Fenton chemistry [45]. If [^•^*OH*] levels rise above baseline conditions, the mtDNA damaged rate is increased and its content reduced. The third and last term represents direct damage to the mtDNA by DOX and was considered to be proportional to the mtDNA content and the drug concentration. The model parameters were fitted using data from in vivo experiments [15, 21] where mtDNA content reductions were measured after treating rats with seven weekly doses of DOX. An extended description of the model’s assumptions, parameters fitting and sensitivities can be found in section S3 of the supplemental material.

## Supporting Information

S1 Text Supplementary information and figures. This document contains all the supplementary information and figures associated with the main text.
(PDF)
