# Supplementary material for: A Biophysical Systems Approach to Identifying the Pathways of Acute and Chronic Doxorubicin Mitochondrial Cardiotoxicity
Source: PLoS Comput Biol. 2016 Nov 21;12(11):e1005214. doi: 10.1371/journal.pcbi.1005214 (PMC5117565; doi:10.1371/journal.pcbi.1005214)
Supplement: S1 Text — This document contains all the supplementary information and figures associated with the main text. (PDF) [file pcbi.1005214.s001.pdf]

Supporting information:
"A Biophysical Systems Approach to Identifying the Pathways of Acute and Chronic Doxorubicin Mitochondrial Cardiotoxicity"

Bernardo L. de Oliveira[1], Steven Niederer[1*]

[1]Department of Biomedical Engineering, Division of Imaging Sciences and Biomedical Engineering, King's College London, London, UK

In this supplemental material we will list and discuss the equations and parameters that were altered in our work and the new models and equations proposed here. For a complete list with all equations and parameters of the original mitochondrial model adapted in this work, we refer to its original publications [1, 2].

S1 ETC Inhibition

To model ETC inhibition by DOX, dose dependent Hill functions were constructed for each of the ETC complexes using IC_{50} values reported in the literature [3]. The Hill coeficient was determined by digitizing the figures in [3], fitting a Hill function to the data obtained and rounding to the nearest integer. The obtained value was $nH = 3$. Section S1.1 presents tests varying this parameter. These functions, depicted in Figure S1, were used to scale the activity of the ETC:

$$C1_{inhib} = 1 - \frac{[DOX]^3}{[DOX]^3 + (400e^{-6})^3} \tag{1}$$

$$C2_{inhib} = 1 - \frac{[DOX]^3}{[DOX]^3 + (2000e^{-6})^3} \tag{2}$$

$$C3_{inhib} = 1 - \frac{[DOX]^3}{[DOX]^3 + (185e^{-6})^3} \tag{3}$$

$$C4_{inhib} = 1 - \frac{[DOX]^3}{[DOX]^3 + (165e^{-6})^3} \tag{4}$$

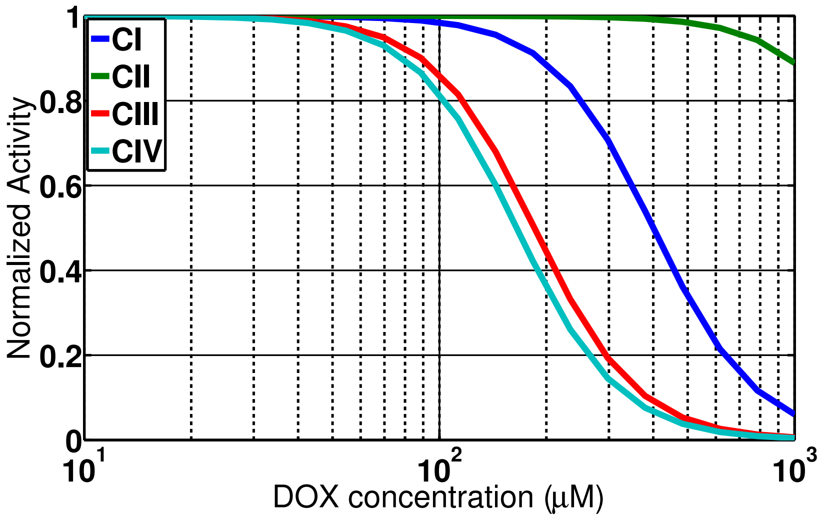

Figure S1: **Dose dependent inhibition of the ETC Complexes by DOX.**

Where $[DOX]$ is the DOX concentration in μM. Following the ETC inhibition strategy dicussed in section S1.2, these inhibition functions were introduced in the ETC Equations 31, 32, 53, 72, 73, 78, 79 and 94 of the mitochondrial model listed in section S4.2.

S1.1 Variations in nH

The Hill coeficient, nH, presented in Section S1, was adjusted based on digitized data from the figures of [3] and rounded to the nearest integer. Here, tests were performed to see the effects of variations in this parameter on the results. Figure S2 presents the acute effects of ETC inhibition assuming different values for nH. In this Figure we can observe that the results present the same qualitative behaviour with a small variation in the concentration in which the mitochondrial function collapses. For the simulations of the chronic effects of DOX, as the concentrations were up to $30\mu M$, which is much lower than the reported IC_{50} values for the ETC, the results were unaffected by variations in nH. Therefore, different choices for the value of this parameter do not alter the conclusions of this study.

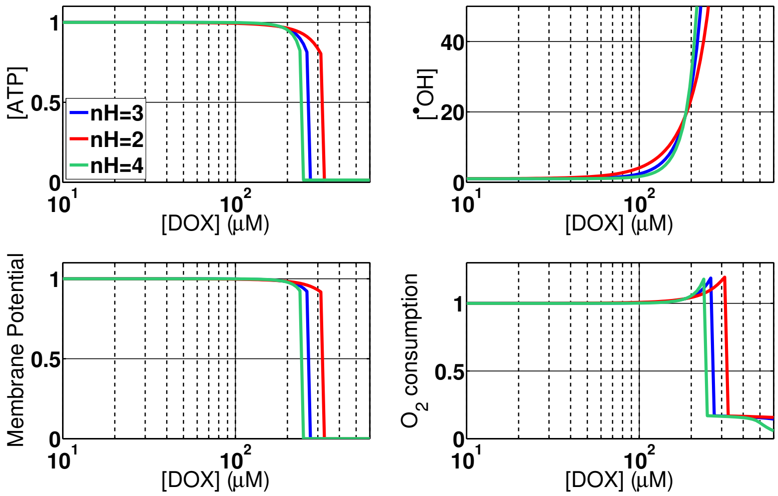

Figure S2: **Simulated effects of ETC inhibition taking into account different values for nH.**

S1.2 Strategies for ETC inhibition

Two different strategies were tested to model ETC inhibition. Strategy 1 was inherited from the original mitochondrial model adopted [2]. This strategy involves ETC inhibition by using the functions described by equations 1 to 4, to scale specific rate constants in the model. Strategy 2, was to inhibit the ETC by using the functions described by equations 1 to 4, to scale the total activity of each complex of the ETC. Both strategies generated qualitively similar results, as depicted in Figure S3. For the simulations of the chronic effects of DOX, as the concentrations were up to $30\mu M$, which is much lower than the reported IC_{50} values for the ETC, the results were unaffected by the choice of ETC inhibition strategy. Therefore, the choice of ETC inhibition strategies do not alter the conclusions of this study. So, all other results in this study were generated with strategy 1, to remain consistent with the adopted mitochondrial model.

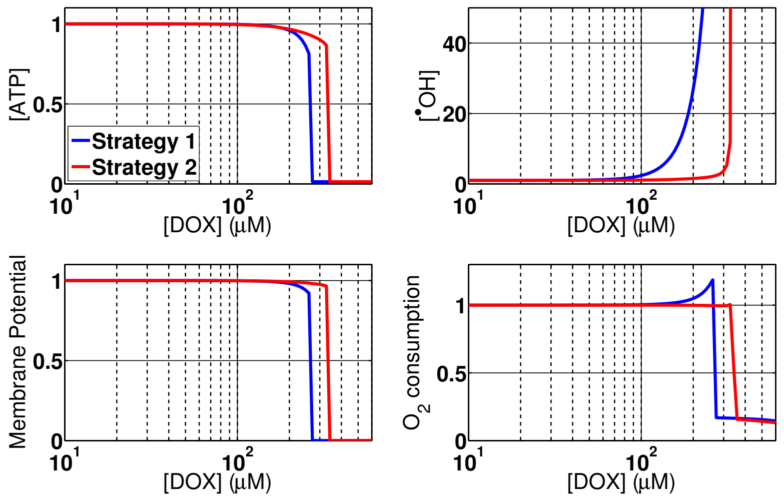

Figure S3: **Simulated effects of ETC inhibition taking into account different strategies.**

S2 Redox Cycling

In our model redox cycling was considered to be proportional to the concentraion of DOX in the mitochondria. This effect was incorporated in the model by increasing the O_2^{-} production in Complex I, which has been identified as the redox cycling site for DOX [4]:

$$a_{42} = (1 + \mathbf{k_{RC}} \cdot [\mathbf{DOX}]) \cdot a_{42}^{*} \cdot O_2$$

Where a_{42} is the rate of $O_2^{\cdot -}$ production by Complex I, $[DOX]$ is the DOX concentration in μM and k_{RC} is a redox cycling constant. A complete list with all equations of the ETC model adopted in this work can be found in the end of this supplemetnal material.

The redox cycling constant, $k_{RC} = 1/15 \mu M^{-1}$, was adjusted using experimental data from Group E of [5] which is the only group in which measurements were made while the drug was still present in the system. This experiment showed a 7% increase in the $O_2^{\cdot -}$ concentration in rats 2 hours after treatment with $1 mg/kg$ of DOX , which is the equivalent of a $30 \mu M$ dose in our model. The experimental data used for this adjustment can be observed in red in Figure S4, where the blue curve is our model prediction for the change in $O_2^{\cdot -}$ concentration for the same protocol.

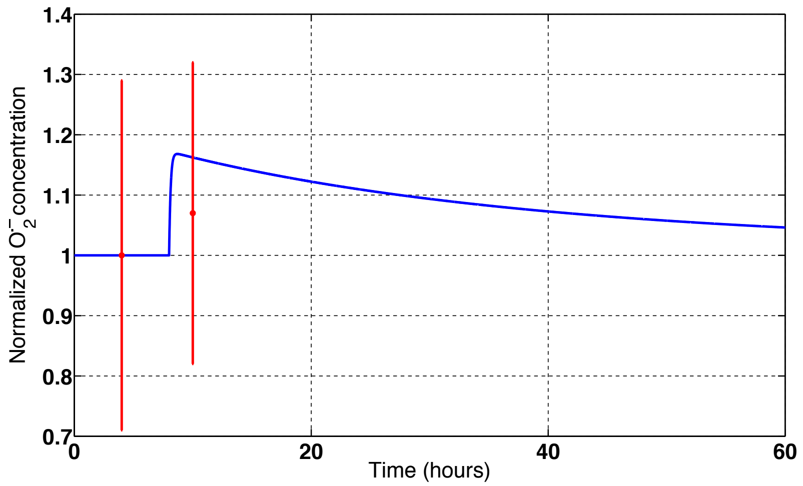

Figure S4: **Superoxide concentration variation for a single therapeutic DOX dose. The red errorbars are experimental measurements of superoxide concentration for untretead rats and 2h after administration of 1mg/kg of DOX.**

S3 MtDNA model

S3.1 Protein expression

In our model, the expression of the mtDNA encoded proteins, mt_{prot}, was considered to be proportional to the mtDNA content. The protein densities from the original model, ρ_{C1}, $C3_{tot}$, $C4_{tot}$ and ρ_{F1}, corresponding to Complex I, III, IV and ATP synthase respectively, were scaled using a function to relate mt_{prot} and mtDNA content:

$$mt_{prot} = \frac{k_b \cdot mtDNA}{mtDNA + k_{prot}} \tag{5}$$

The constants, $k_b = 1.42$ and $k_{prot} = 0.32$, were fitted to experimental data [5] where paired measurements following exposure to DOX, showed a correlation between the mtDNA content and the activity of mtDNA encoded proteins, as shown in Figure S5.

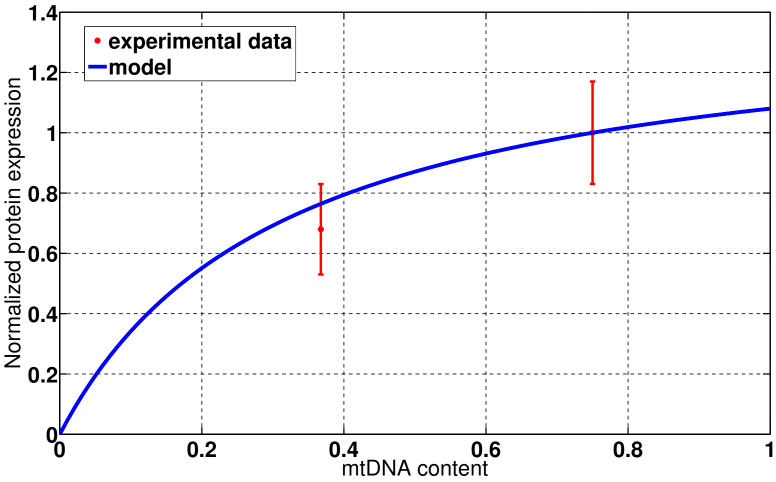

Figure S5: **Relationhip between mtDNA content and mtDNA encoded proteins expression in our model [5].**

S3.2 Fitting mtDNA damage and repair model

The four parameters in the equation for the mtDNA content time derivative, repeated here for convenience, were adjusted using experimental data and constraint to the baseline conditions of the model.

$$\frac{d(mtDNA)}{dt} = \alpha \cdot \left(\frac{(1 - mtDNA)}{(1 - mtDNA) + \kappa} \right) - \beta \cdot [^{\bullet}OH]_n \cdot mtDNA - \gamma[DOX] \cdot mtDNA. \tag{6}$$

The function that defines the phase plot of mtDNA in the absence of DOX, depicted in the top panel of Figure 4 is:

$$f(mtDNA) = \alpha \cdot \left(\frac{(1 - mtDNA)}{(1 - mtDNA) + \kappa} \right) - \beta \cdot [^{\bullet}OH]_n \cdot mtDNA \tag{7}$$

The first assumption is that the model is in steady state at baseline conditions. To enforce this, we have $f(mtDNA_b) = 0$, where $mtDNA_b$ is the mtDNA content at baseline conditions. As $[^{\bullet}OH]_n = 1$ at baseline conditions, from Equation 7 we get a hard constrain for one of the parameters:

$$\beta = \frac{\alpha}{mtDNA_b} \cdot \left(\frac{(1 - mtDNA_b)}{(1 - mtDNA_b) + \kappa} \right) \tag{8}$$

Differentiating equation 7 we obtain:

$$\frac{df(mtDNA)}{dmtDNA} = \frac{-\alpha \cdot \kappa}{(1 - mtDNA + \kappa)^2} - \beta \cdot [^{\bullet}OH]_n - \beta \cdot mtDNA \cdot \frac{d[^{\bullet}OH]_n}{dmtDNA} \tag{9}$$

The second assumption is that the mtDNA content is in a stable steady state at baseline. To enforce a this, we set:

$$\frac{df(mtDNA_b)}{dmtDNA} < 0 \tag{10}$$

Combining Equations 9 and 10 we get:

$$\frac{-\alpha \cdot \kappa}{(1 - mtDNA_b + \kappa)^2} - \beta - \beta \cdot mtDNA_b \cdot \frac{d[^{\bullet}OH]_n}{dmtDNA} < 0 \tag{11}$$

Combining Equations 8 and 11 and simplifying we get:

$$\frac{-\kappa}{(1 - mtDNA_b + \kappa)} - \frac{1 - mtDNA_b}{mtDNA_b} - (1 - mtDNA_b) \cdot \frac{d[^{\bullet}OH]_n}{dmtDNA} < 0 \tag{12}$$

By assuming $mtDNA_b = 0.75$ and numerically calculating the hydroxil radical concentration derivative with respect to mtDNA at that point, we get the following soft constraint:

$$\kappa \gtrsim 0.039 \tag{13}$$

After these constraints were applied, a parameter sweep was performed in order to find the parameter set that minimizes the the error between the model predictions and experimental data. The error associated to a parameter set was calculated using the L^2-norm:

$$e = \sqrt{(x_{1,sim} - x_{1,exp})^2 + (x_{2,sim} - x_{2,exp})^2 + (x_{3,sim} - x_{3,exp})^2} \tag{14}$$

Where $x_{1,exp}$ and $x_{2,exp}$ are the data points corresponding to Group B and C from [5] respectively and $x_{3,exp}$ corresponds to Group B from [6]. To calculate the corresponding simulated values, $x_{1,sim}$, $x_{2,sim}$ and $x_{3,sim}$, simulations were performed replicating the exprimental protocols of each of the experimental data points. The lower bound for κ is defined in Equation 13 and for α and γ, it was zero by definition. The upper bound for κ was 0.16 as the results presented low sensitivity for values greater than that, with the error monotonically increasing. The upper bounds for α and γ were $1e^{-10}$ and $3e^{-5}$ respectively, as simulations with values greater than those, generated no results within the reported experimental errorbars. Figure S6 shows the results for all simulated parameter sets, where the colorbar represent the error and the white areas represent parameter sets that generated results outside of the reported experimental errorbars. Table S1 presents the optimal parameter set found which generated an error of e=0.1595. It is possible to observe in Figure S6 that all parameter sets that

matched experimental data present a value for γ equal or greater than the value of the parameter set used in the study. Therefore, for all of these parameter sets, direct damage to mtDNA by DOX is the primary toxicity pathway responsible for triggering the vicious cycle that leads to mitochondrial dysfucntion as concluded in our study.

The sensitivity of the error with respect to the parameters was calculated using Equation 15.

$$S_i = \left| \frac{e(p_i + 0.01 \cdot p_i) - e(p_i)}{0.01 \cdot e(p_i)} \right| \tag{15}$$

where S_i is the sensitivity of the error with respect to the parameter p_i.

Table S1: **Parameters for the mtDNA derivative equation**

Parameter	Value	Unit	Sensitivity
α	$5.625 \cdot 10^{-11}$	ms^{-1}	0.0024
β	$6.94 \cdot 10^{-11}$	ms^{-1}	0.0024
γ	$8.1 \cdot 10^{-6}$	$ms^{-1}M^{-1}$	0.0023
κ	$4.5 \cdot 10^{-2}$	Unitless	0.0019

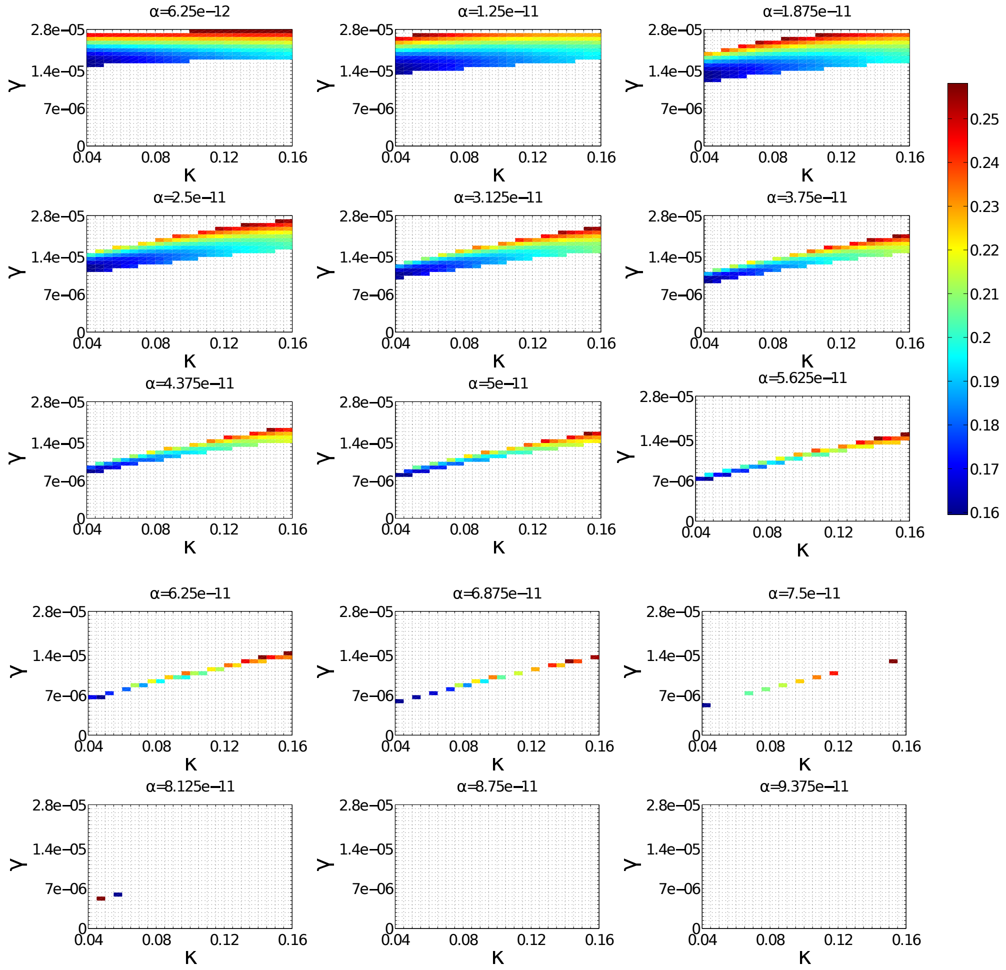

Figure S6: L^2**norm errors of the parameter sets tested. White areas correspond to parameter sets which generated results outside of the errorbars in at least one point.**

S3.3 Hydroxil radical production

This section describes how the normalized concentration of hydroxil radical, $[{}^\bullet OH]_n$, in Equation 6 is calculated in the model. The model assumes that hydroxyl radical is produced in a two step iron catalyzed Haber-Weiss reaction. In the first step of the Haber-Weiss reaction, Equation 16, superoxide oxidizes proteins containing Fe^{3+} in iron-sulfur clusters, inactivating these proteins and releasing Fe^{2+} [7]. In the second step of the Haber-Weiss reaction, Equation 17, also known as Fenton's reaction, the free Fe^{2+} then reacts with peroxide, producing hydroxil radical:

$$Fe^{3+} + O_2^{\cdot -} \rightarrow Fe^{2+} + O_2 \tag{16}$$

$$Fe^{2+} + H_2O_2 \rightarrow Fe^{3+} + OH^- + {}^\bullet OH \tag{17}$$

The model assumes that the concentrations of O_2, and of the catalyzer Fe^{3+} are constant, as these concentrations are much greater than the concentration of superoxide. Therefore, the hydroxil radical production can be calculated using the net reaction, without explicitly calculating the concentration of the intermediate reactant Fe^{2+}:

$$O_2^{\cdot -} + H_2O_2 \rightarrow O_2 + OH^- + {}^\bullet OH \tag{18}$$

From Eq 18, the rate of hydroxyl radical production, $j_{p^\bullet OH}$ is:

$$j_{p^\bullet OH} = k_p \cdot [O_2^{\cdot -}][H_2O_2] \tag{19}$$

Hydroxil radical is very reactive and upon formation quickly reacts indiscriminately with substances within a 93Å radius with a half-life of $10^{-9}s$ [8]. Therefore, the model assumes that the concentration of hydroxil radical concentration is in steady state, i.e. the rate of production, $j_{p^\bullet OH}$, is equal to the rate of consumption of hydroxil radical $j_{c^\bullet OH}$:

$$j_{p^\bullet OH} \simeq j_{c^\bullet OH} = k_c \cdot [{}^\bullet OH][T] \tag{20}$$

Where [T] is the combined concentraion of all molecules that are targets of hydroxil radical oxidation, which include multiple proteins and lipids. As the concentration of hydroxil radical is much less than the combined concentration of oxidation targets, $[{}^\bullet OH] \ll [T]$, we assume that [T] is constant and that the consumption of hydroxil radical is directly proportional to its concentration:

$$j_{p^\bullet OH} \simeq k_2 \cdot [{}^\bullet OH] \tag{21}$$

Where $k_2 = k_c \cdot [T]$. Substituting Equation 21 in Equation 19 and normalizing with respect to baseline conditions we get:

$$[{}^\bullet OH]_n = \frac{[{}^\bullet OH]}{[{}^\bullet OH]_b} \simeq \frac{[O_2^{\cdot -}][H_2O_2]}{[O_2^{\cdot -}]_b[H_2O_2]_b} \tag{22}$$

This concentration, $[{}^\bullet OH]$, is then used in Equation 6 to calculate the term related to oxidative damage to mtDNA.

S3.4 Iron Chelating therapy

The co-administration of dexrazoxane along with DOX has been shown to prevent the rise in free iron levels observed when administring DOX in isolation [9]. Therefore, in this study, a simplified model for the effect of dexrazoxane co-treatment is adopted by assuming that the concentration of Fe^{2+} is kept at baseline during the iron chelating treatment. So, during dexrazoxane treatment, from Equation 17 we get:

$$j_{p^\bullet OH} = k_p \cdot [Fe^{2+}]_b [H_2O_2] \tag{23}$$

Following the same steps from section S3.3, we get that during dexrazoxane treatment the hydroxil radical concentratil is given by:

$$[^\bullet OH]_n = \frac{[^\bullet OH]}{[^\bullet OH]_b} \simeq \frac{[H_2O_2]}{[H_2O_2]_b} \tag{24}$$

S4 Model Description

S4.1 Parameters

All simulations in this work consider that the mitochondria are in state 3 respiration with the following input parameters:

Table S2: **Input parameters for the model**

Parameter	Value	Unit
$[H^+]_i$	1×10^{-4}	mM
$[Na^+]_i$	5	mM
$[Ca^{2+}]_i$	1×10^{-4}	mM
$[ADP]_i$	1	mM
$[Pi]_i$	3	mM
pH_i	7	Unitless
$[GLU]$	30	mM
$[AcCoA]$	0.1	mM

S4.2 ETC Equations

Figure S7 shows a schematic of the ETC model used in this work. The equations that describe this ETC model are reproduced here, where the alterations made in this work are highlighted in bold font. For a complete list with all equations and parameters of the original mitochondrial model adapted in this work, we refer to its original publications [1, 2].
Complex I:
Transition rates between states:

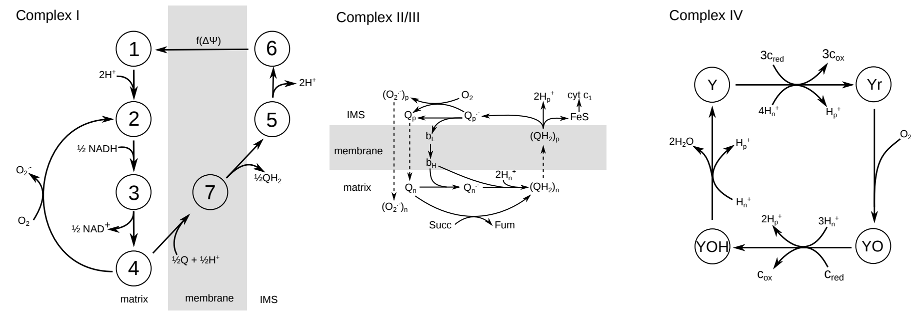

Figure S7: **Reaction schemes for the ETC model adopted.**

$$a_{12} = a^*_{12} \cdot H_m^2 \tag{25}$$

$$a_{65} = a^*_{65} \cdot H_i^2 \tag{26}$$

$$a_{61} = a^*_{61} \cdot e^{-(\Delta\Psi - \Delta\Psi_B)\cdot \frac{F}{RT}} \tag{27}$$

$$a_{16} = a^*_{16} \cdot e^{(\Delta\Psi - \Delta\Psi_B)\cdot \frac{F}{RT}} \tag{28}$$

$$a_{23} = a^*_{23} \cdot NADH^{1/2} \tag{29}$$

$$a_{43} = a^*_{43} \cdot (NAD^+)^{1/2} \tag{30}$$

$$a_{47} = a^*_{47} \cdot Q^{1/2} \cdot H_m^{1/2} \cdot \mathbf{C1_{inhib}} \tag{31}$$

$$a_{57} = a^*_{57} \cdot (QH_2)^{1/2} \cdot \mathbf{C1_{inhib}} \tag{32}$$

$$a_{42} = (1 + \mathbf{k_{RC}} \cdot [\mathbf{DOX}]) \cdot a^*_{42} \cdot O_2 \tag{33}$$

$$a_{24} = a^*_{24} \cdot O_2^{\cdot -} \cdot e^{((E_{O_2^{\cdot -}} - E_{FMN})\cdot \frac{F}{RT}} \tag{34}$$

Where a_{xy} is the transition rate from state x to y. Superoxide production by Complex I involves a single electron reaction, in the original mitochondria model this was represented as a two electron reaction. We have updated this reaction, represented in Equation 34, to reflect this.

Flux equations:

$$J_{res} = 60 \cdot \rho_{C1} \cdot \mathbf{mt_{prot}} \cdot (F_4 \cdot a_{47} - F_7 \cdot a_{74}) \tag{35}$$

$$v_{c1} = J_{res}/2 \tag{36}$$

$$VROS_{c1} = 60 \cdot \rho_{C1} \cdot \mathbf{mt_{prot}} \cdot (F_4 \cdot a_{42} - F_2 \cdot a_{24}) \tag{37}$$

Where F_x is the occupancy probability of state x.

Complex II/III:

System of differential equations describing complex III:

$$\frac{dQ_n}{dt} = v_5 - v_{7\_bLox} - v_{7\_bLred} - v_1 \tag{38}$$

$$\frac{dQ_n^{\cdot -}}{dt} = v_{7\_bLox} + v_{7\_bLred} - v_{8\_bLox} - v_{8\_bLred} \tag{39}$$

$$\frac{d(QH_2)_n}{dt} = v_{8\_bLox} + v_{8\_bLred} + v_1 - v_2 \tag{40}$$

$$\frac{d(QH_2)_p}{dt} = v_2 - v_3 \tag{41}$$

$$\frac{dQ_p^{\cdot -}}{dt} = v_3 - v_{10} - v_{10b} - v_{4\_bHox} - v_{4\_bHred} \tag{42}$$

$$\frac{dQ_p}{dt} = v_{10} + v_{10b} + v_{4\_bHox} + v_{4\_bHred} - v_5 \tag{43}$$

$$\frac{db1}{dt} = v_{7\_bLox} + v_{8\_bLox} - v_{4\_bHox} \tag{44}$$

$$\frac{db2}{dt} = v_{4\_bHox} + v_{7\_bLred} + v_{8\_bLred} - v_6 \tag{45}$$

$$\frac{db3}{dt} = -v_{4\_bHred} + v_6 - v_{7\_bLox} - v_{8\_bLox} \tag{46}$$

$$\frac{db4}{dt} = v_{4\_bHred} - v_{7\_bLred} - v_{8\_bLred} \tag{47}$$

$$\frac{dFeS_{ox}}{dt} = v_9 - v_3 \tag{48}$$

$$\frac{dcytc1_{ox}}{dt} = v_{33} - v_9 \tag{49}$$

$$\frac{dcytc_{ox}}{dt} = Ve - v_{33} \tag{50}$$

$$\frac{d\Delta\Psi}{dt} = (VhRes - Vhu - Vhleak)/C_{mito} \tag{51}$$

Where b_1 represents the b-heme state where b_L and b_H are both oxidized; b_2 is b_L reduced, b_H oxidized; b_3 is b_L oxidized, b_H reduced; b_4 is both reduced.
Rate equations for complexes II and III:
Reaction 1 - reduction of Q to QH_2:

$$v_1 = v_{c1} + v_{c2} \tag{52}$$

(see v_{c1} above)

$$v_{c2} = \frac{V_{SDH} \cdot Q_n/(Q_n + (QH_2)_n)}{K_m + Q_n/(Q_n + (QH_2)_n)} \cdot \mathbf{C2_{inhib}} \tag{53}$$

Reaction 2 - diffusion of QH_2 across the membrane:

$$v_2 = k_d \cdot ((QH_2)_n - (QH_2)_p) \tag{54}$$

Reaction 3 - QH_2 to FeS e^- transfer:

$$k_3 = k_{03} \cdot Keq3 \cdot e^{(2.3\cdot(7-pH_p))} \tag{55}$$
$$k_{-3} = k_{03} \tag{56}$$
$$v_3 = k_3 \cdot (QH_2)_p \cdot FeS_{ox} - k_{-3} \cdot Q_p^{\cdot-} \cdot FeS_{red} \tag{57}$$

Reaction 4 - $Q_p^{\cdot-}$ to b_H e^- transfer:

$$k_{4\_bHox} = k_{04} \cdot Keq4\_bHox \cdot e^{(-\alpha\cdot\delta_1\cdot F/(RT)\cdot\Delta\Psi)} \tag{58}$$
$$k_{4\_bHred} = k_{04} \cdot Keq4\_bHred \cdot e^{(-\alpha\cdot\delta_1 F/(RT)\cdot\Delta\Psi)} \tag{59}$$
$$k_{-4\_bHox} = k_{04} \cdot e^{(\alpha\cdot(1-\delta_1)/(RT)\cdot\Delta\Psi)} \tag{60}$$
$$k_{-4\_bHred} = k_{04} \cdot e^{(\alpha\cdot(1-\delta_1)\cdot F/(RT)\cdot\Delta\Psi)} \tag{61}$$
$$v_{4\_bHox} = k_{4\_bHox} \cdot Q_p^{\cdot-} \cdot b1 - k_{-4\_bHox} \cdot Q_p \cdot b_2 \tag{62}$$
$$v_{4\_bHred} = k_{4\_bHred} \cdot Q_p^{\cdot-} \cdot b_3 - k_{-4\_bHred} \cdot Q_p \cdot b_4 2 \tag{63}$$

Reaction 5 - diffusion of Q across the membrane

$$v_5 = k_d \cdot (Q_p - Q_n) \tag{64}$$

Reaction 6 - b_H to b_L e^- transfer

$$k_6 = k_{06} \cdot Keq6 \cdot e^{(-\beta\cdot\delta_2\cdot F/(RT)\cdot\Delta\Psi)} \tag{65}$$
$$k_{-6} = k_{06} \cdot e^{(\beta\cdot(1-\delta_2)/(RT)\cdot\Delta\Psi)} \tag{66}$$
$$v_6 = k_6 \cdot b_2 - k_{-6} \cdot b_3 \tag{67}$$

Reaction 7 - b_L to Q_n e^- transfer

$$k_{7\_bLox} = k07\_bLox \cdot Keq7\_bLox \cdot e^{(-\gamma\cdot\delta_3\cdot F/(RT)\cdot\Delta\Psi)} \tag{68}$$
$$k_{7\_bLred} = k07\_bLred \cdot Keq7\_bLred \cdot e^{(-\gamma\cdot\delta_3\cdot F/(RT)\cdot\Delta\Psi)} \tag{69}$$
$$k_{-7\_bLox} = k07\_bLox \cdot e^{(\gamma\cdot(1-\delta_3)\cdot F/(RT)\cdot\Delta\Psi)} \tag{70}$$
$$k_{-7\_bLred} = k07\_bLred \cdot e^{(\gamma\cdot(1-\delta_3)/(RT)\cdot\Delta\Psi)} \tag{71}$$
$$v_{7\_bLox} = (k_{7\_bLox} \cdot Q_n \cdot b_3 - k_{-7\_bLox} \cdot Q_n^{\cdot-} \cdot b1) \cdot \mathbf{C3_{inhib}} \tag{72}$$
$$v_{7\_bLred} = (k_{7\_bLred} \cdot Q_n \cdot b4 - k_{-7\_bLred} \cdot Q_n^{\cdot-} \cdot b2) \cdot \mathbf{C3_{inhib}} \tag{73}$$

Reaction 8 - b_L to $Q_n^{\cdot-}$ e^- and proton transfer:

$$k_{8\_bLox} = k08\_bLox \cdot Keq8\_bLox \cdot e^{(-\gamma\cdot\delta_3\cdot F/(RT)\cdot\Delta\Psi)} \cdot e^{(2.3\cdot2\cdot(7-pH_n))} \tag{74}$$
$$k_{8\_bLred} = k08\_bLred \cdot Keq8\_bLred \cdot e^{(-\gamma\cdot\delta_3\cdot F/(RT)\cdot\Delta\Psi)} \cdot e^{(2.3\cdot2\cdot(7-pH_n))} \tag{75}$$
$$k_{-8\_bLox} = k08\_bLox \cdot e^{(\gamma\cdot(1-\delta_3)/(RT)\cdot\Delta\Psi)} \tag{76}$$
$$k_{-8\_bLred} = k08\_bLred \cdot e^{(\gamma\cdot(1-\delta_3)\cdot F/(RT)\cdot\Delta\Psi)} \tag{77}$$
$$v_{8\_bLox} = (k_{8\_bLox} \cdot Q_n^{\cdot-} \cdot b3 - k_{-8\_bLox} \cdot (QH_2)_n \cdot b1) \cdot \mathbf{C3_{inhib}} \tag{78}$$
$$v_{8\_bLred} = (k_{8\_bLred} \cdot Q_n^{\cdot-} \cdot b4 - k_{-8\_bLred} \cdot (QH_2)_n \cdot b2) \cdot \mathbf{C3_{inhib}} \tag{79}$$

Reaction 9 - FeS to cyt c_1 e^- transfer

$$k_9 = k09 \cdot Keq9 \tag{80}$$
$$k_{-9} = k09 \tag{81}$$
$$v_9 = k_9 \cdot FeS_{red} \cdot cytc1_{ox} - k_{-9} \cdot FeS_{ox} \cdot cytc1_{red} \tag{82}$$

Reaction 10 - superoxide production from $Q_p^{\cdot -}$

$$k_{10} = k010 \cdot Keq10 \tag{83}$$
$$k_{-10} = k010 \tag{84}$$
$$v_{10} = k_{10} \cdot Q_p^{\cdot -} \cdot O_2 - k_{-10} \cdot Q_p \cdot O_2^{\cdot -} \tag{85}$$
$$v_{10b} = k_{10} \cdot Q_p^{\cdot -} \cdot O_2 - k_{-10} \cdot Q_p \cdot O_2^{\cdot -} \tag{86}$$

Reaction 33 - cyt c_1 to cyt c e^- transfer

$$v_{33} = k_{33} \cdot cytc1_{red} \cdot cytc_{ox} - k_{-33} \cdot cytc1_{ox} \cdot cytc_{red} \tag{87}$$

Conservation relations

$$FeS_{red} = C3_{tot} \cdot \mathbf{mt_{prot}} - FeS_{ox} \tag{88}$$
$$cytc1_{red} = C3_{tot} \cdot \mathbf{mt_{prot}} - cytc1_{ox} \tag{89}$$
$$cytc_{red} = C4_{tot} \cdot \mathbf{mt_{prot}} - cytc_{ox} \tag{90}$$

Comple IV:

Rate equations used in complex IV model:

$$a_{12} = k_{34} \cdot e^{-d_5 \cdot 4 \cdot \Delta\Psi \cdot F/RT} \cdot cytc_{red}^3 \cdot H_n^4 \tag{91}$$
$$a_{14} = k_{-37} \cdot e^{(1-d_5) \cdot \Delta\Psi \cdot F/RT} \cdot H_p \tag{92}$$
$$a_{21} = k_{-34} \cdot e^{(1-d_5) \cdot 4 \cdot \Delta\Psi \cdot F/RT} \cdot cytc_{ox}^3 \cdot H_p \tag{93}$$
$$a_{23} = k_{35} \cdot O_2 \cdot \mathbf{C4_{inhib}} \tag{94}$$
$$a_{34} = k_{36} \cdot e^{-d_5 \cdot 3 \cdot \Delta\Psi \cdot F/RT} cytc_{red} \cdot H_n^3 \tag{95}$$
$$a_{41} = k_{37} \cdot e^{-d_5 \cdot \Delta\Psi \cdot F/RT} \cdot H_n \tag{96}$$
$$a_{43} = k_{-36} \cdot e^{(1-d_5) \cdot 3 \cdot \Delta\Psi \cdot F/RT} \cdot cytc_{ox} \cdot H_p^2 \tag{97}$$

Flux equations:

$$v_{34} = C4_{tot} \cdot \mathbf{mt_{prot}} \cdot (Y \cdot a_{12} - Yr \cdot a_{21}) \tag{98}$$
$$v_{35} = C4_{tot} \cdot \mathbf{mt_{prot}} \cdot Yr \cdot a_{23} \tag{99}$$
$$v_{36} = C4_{tot} \cdot \mathbf{mt_{prot}} \cdot (a_{34} \cdot YO - a_{43} \cdot YOH) \tag{100}$$
$$v_{37} = C4_{tot} \cdot \mathbf{mt_{prot}} \cdot (a_{41} \cdot YOH - a_{14} \cdot Y) \tag{101}$$
$$V_e = 3v_{34} + v_{35} \tag{102}$$
$$V_H = 4v_{34} + 3v_{36} + v_{37} \tag{103}$$
$$V_{O2} = v_{35} \tag{104}$$
$$VhRes = 2v_3 + V_H \tag{105}$$

ATP generation and proton leak systems:

$$\Delta pH = pH_p - pH_n \tag{106}$$

$$\mu H = -2.303 \cdot RT/F \cdot \Delta pH + \Delta\Psi \tag{107}$$

$$V_{AF1} = \frac{K_{ATPase\_app} \cdot ATP}{P_i \cdot (ADP^{3+} + HADP)} \tag{108}$$

$$Vhu = -\rho_{F1} \cdot \mathbf{mt_{prot}} \cdot \frac{300 \cdot p_a + 300 \cdot p_a \cdot V_{AF1} - 3e^{3\cdot\mu_H \cdot F/RT} \cdot (p_a + p_b)}{e^{150F/RT} + p_1 \cdot e^{150F/RT} \cdot V_{AF1} + e^{3\cdot\mu_H \cdot F/RT} \cdot (p_2 + p_3 \cdot V_{AF1})} \tag{109}$$

$$Vhleak = gH \cdot \mu_H \tag{110}$$

References

[1] Gauthier LD, Greenstein JL, Cortassa S, O'Rourke B, Winslow RL. A Computational Model of Reactive Oxygen Species and Redox Balance in Cardiac Mitochondria. Biophysical Journal. 2013;105:1045–1056.

[2] Gauthier LD, Greenstein JL, O'Rourke B, Winslow RL. An Integrated Mitochondrial ROS Production and Scavenging Model: Implications for Heart Failure. Biophysical Journal. 2013;105(12):2832 – 2842.

[3] Nicolay K, de Kruijff B. Effects of adriamycin on respiratory chain activities in mitochondria from rat liver, rat heart and bovine heart. Evidence for a preferential inhibition of complex III and IV. Biochimica et Biophysica Acta. 1987;892:320–330.

[4] Davies KJA, Doroshow JH. Redox cycling of anthracyclines by cardiac mitochondria. I. Anthracycline radical formation by NADH dehydrogenase. The Journal of Biological Chemestry. 1986;261(7):3060–7.

[5] Lebrecht D, Setzer B, Ketelsen UP, Haberstroh J, Walker UA. Time-dependent and tissue-specific accumulation of mtDNA and respiratory chain defects in chronic doxorubicin cardiomyopathy. Circulation. 2003;108(19):2423–9.

[6] Lebrecht D, Geist A, Ketelsen UP, Haberstroh J, Setzer B, Walker U. Dexrazoxane prevents doxorubicin-induced long-term cardiotoxicity and protects myocardial mitochondria from genetic and functional lesions in rats. British Journal of Pharmacology. 2007;151:771–778.

[7] Vasquez-Vivar J, Kalyanaraman B, Kennedy MC. Mitochondrial Aconitase Is a Source of Hydroxyl Radical. The Journal of Biological Chemestry. 2000;275:14064 –14069.

[8] Thomas C, Mackey MM, Diaz AA, Cox DP. Hydroxyl radical is produced via the Fenton reaction in submitochondrial particles under oxidative stress: implications for diseases associated with iron accumulation. Redox Report. 2013;14(3):102–108.

[9] Ichikawa Y, Ghanefar M, Bayeva M, Wu R, Khechaduri A, Prasad SVN, et al. Cardiotoxicity of doxorubicin is mediated through mitochondrial iron accumulation. Journal of Clinical Investigation. 2014;124(2):617–630.
